# Supplementary material for: Effect of implementing of the IDEAL discharge model on satisfaction of patient referred to trauma emergency department
Source: PLoS One. 2024 Jun 13;19(6):e0304969. doi: 10.1371/journal.pone.0304969 (PMC11175502; doi:10.1371/journal.pone.0304969)
Supplement: S1 File — (DOCX) [file pone.0304969.s003.docx]

| **Items** | **CVI** | **CVR** | | | |
| --- | --- | --- | --- | --- | --- |
|  |  | Ambiguity | simplicity | relevance | |
| **On arrival at the emergency department** | | | | |  |
| 1. Was the department explained to you upon arrival? | 0.9 | 0.93 | 0.88 | 0.86 | |
| 1. Were you delayed when you arrived for a visit? | 0.85 | 0.85 | 0.9 | 1 | |
| 1. Was the purpose of the examination explained to you upon arrival? | 0.88 | 0.88 | 0.88 | 0.86 | |
| 1. Was the purpose of care explained to you upon arrival? | 0.78 | 0.78 | 0.93 | 0.86 | |
| 1. Were people and departments introduced to you upon arrival? | 0.93 | 0.88 | 0.85 | 1 | |
| **During hospitalization in the emergency department** | | | | |  |
| 1. Were the examination results explained to you during hospitalization? | 0.88 | 0.85 | 0.78 | 1 | |
| 1. Before doing any procedure, was it explained to you regarding care and procedures during hospitalization? | 0.9 | 0.88 | 0.93 | 1 | |
| 1. Did you have unanswered questions during hospitalization? | 0.93 | 0.78 | 0.88 | 0.86 | |
| **Discharge time from the emergency department** | | | | |  |
| 1. Was it explained to you about self-care at home? | 0.9 | 0.88 | 0.93 | 0.86 | |
| 1. Was the time of the next visit to the doctor explained to you after discharge? | 0.85 | 0.93 | 0.88 | 0.86 | |
| 1. Were you told how to follow up the laboratory results and...? | 0.85 | 0.85 | 0.93 | 0.86 | |
| 1. Did you have unanswered questions at the time of discharge? | 0.9 | 0.88 | 0.85 | 0.86 | |
| **Overall satisfaction with nursing care in the emergency department** | | | | |  |
| 1. Did the nurse have the necessary ability to provide care? | 0.85 | 0.78 | 0.88 | 1 | |
| 1. Was the nurse's behavior respectful? | 0.88 | 0.88 | 0.78 | 1 | |
| 1. Has the nurse given the necessary information about the continuation of the treatment? | 0.78 | 0.78 | 0.93 | 1 | |
| 1. Did the nurse pay the necessary attention to the patients? | 0.93 | 0.93 | 0.93 | 0.86 | |
| **Overall satisfaction with the doctor in the emergency department** | | | | |  |
| 1. Did the doctor give the necessary information about the treatment? | 0.93 | 0.88 | 0.88 | 1 | |
| 1. Was the doctor's behavior respectful? | 0.88 | 0.93 | 0.9 | 0.86 | |
| 1. Was the doctor's explanation about the continuation of the treatment complete? | 0.9 | 0.85 | 0.85 | 0.86 | |
| 1. Did the doctor give you the necessary time? | 0.85 | 0.88 | 0.87 | 0.86 | |
| **Overall patient satisfaction from the emergency department** | | | | |  |
| 1. I recommend this treatment center to others. | 0.85 | 0.88 | 0.88 | 1 | |
| 1. I am satisfied with the services provided in the emergency department. | 0.87 | 0.93 | 0.88 | 1 | |

Result of the quantitative content validity
