# Supplementary material for: Effect of implementing of the IDEAL discharge model on satisfaction of patient referred to trauma emergency department
Source: PLoS One. 2024 Jun 13;19(6):e0304969. doi: 10.1371/journal.pone.0304969 (PMC11175502; doi:10.1371/journal.pone.0304969)
Supplement: S2 File — (PDF) [file pone.0304969.s004.pdf]

RESEARCH PROPOSAL

NON-COMMUNICABLE DISEASES RISK FACTORS AMONG THE STAFF OF UNIVERSITI KEBANGSAAN MALAYSIA AND THE EFFECTIVENESS OF MYMIND AND SLIM SHAPE 2.0 INTERVENTIONS IN IMPROVING THE HEALTH, WELLNESS, AND WORK PRODUCTIVITY

Project leader : Prof. Dr. Mohd Rizal Haji Abdul Manaf

Department: Department of Community Health, Faculty of Medicine, Universiti Kebangsaan Malaysia

Executive summary

This study aims to investigate the non-communicable disease risk factor profile among the staff of the Universiti Kebangsaan Malaysia (UKM) Bangi campus and evaluate the effectiveness of the MyMind and SLIM SHAPE 2.0 intervention programs in reducing cardiovascular disease risk scores, maintaining ideal body weight, improving work productivity and quality of life. This study will be conducted through three phases. Phase 1 involves the baseline data collection on the non-communicable disease risk factor profiles which is done through questionnaires, health examinations, and blood tests. The questionnaire included demographic, socioeconomic, and lifestyle profiles (smoking status, alcohol consumption, body mass index, body composition, physical activity, and dietary practices) and clinical information (co-morbidities and family history of non-communicable diseases). The health examination also involves measuring blood pressure. The blood test assesses the risk profile including blood glucose levels, cholesterol, haemoglobin A1c, kidney and liver function tests, and genetic polymorphisms. Information obtained through questionnaires, health examinations, and blood tests will be able to measure the risk of cardiovascular disease through the Framingham coronary disease prediction score. A total of 1000 academic and non-academic staff will be selected through stratified random sampling. Phase 2 involves intervention activities of dietary, physical activity, and psychological well-being that will be standardized by the Second (MyMind Intervention Program) and the Third Research Project (SLIM SHAPE 2.0 Intervention Program). In addition, there will be a control group that receives standard care interventions from the UKM Health Centre. Staff members with a Body Mass Index of 25 or higher are chosen as subjects for the intervention programs. Phase 3 is the post-intervention evaluation of the effectiveness of the MyMind Intervention Program and SLIM SHAPE 2.0 compared to the control group. This phase will involve data collection as in Phase 1. Assessment of cardiovascular disease risk scores, quality of life, body mass index, and work performance before (Phase 1) and after (Phase 3) intervention activities (Phase 2) will determine the effectiveness of the MyMind and SLIM SHAPE 2.0 Intervention Program. The outcomes of this study are expected to prove that both intervention programs can help the academic and non-academic staff to reduce the risk of non-communicable diseases especially cardiovascular diseases, to maintain an ideal body weight, and to improve their work performance and quality of life in the future. Thus, the MyMind and SLIM SHAPE 2.0 intervention programs can be implemented as an annual program for UKM staff to improve their health, well-being and increase their work productivity.

Literature review

Non-communicable diseases are a group of diseases that involve cardiovascular diseases, chronic respiratory diseases, cancer, diabetes, and mental health. Among these, cardiovascular disease, especially coronary heart disease, contributes to the highest death rate in the world (Murphy et al. 2017) including Malaysia (Khazanah Research Institute 2017).

The rise of non-communicable diseases is said to be similar to a continuous disease attack or a global pandemic (Allen 2017). This occurs as a result of the rising non-communicable disease risk factors in the global population (Foundation PharmAccess 2009). Risk factors such as demographics (Chung et al. 2009; Liu et al. 2017), socioeconomic (Allen et al. 2017), lifestyle (Chakma & Gupta 2017), clinical information (Klemenc-ketis & Peterlin 2013), body mass index (Banjare & Bhalerao 2016), blood pressure (Gupta & Xavier 2018), blood tests (Sardarinia et al. 2016), Framingham coronary heart risk score (Chakma & Gupta 2017) and metabolic syndrome (Perrone-filardi et al. 2015) may increase the risk of non-communicable diseases. Other than that, occupational factors also contribute to an increase in the risk factors for this non-communicable disease (Gebremariam et al. 2018; Mcnamara et al. 2018). However, studies on the risk profile and status of non-communicable diseases involving academic and non-academic university staff in Malaysia are still limited. A more comprehensive knowledge of the risk factors of non-communicable diseases through questionnaires, health examinations, and blood tests would help the university superiors to know the health status of their employees. Early prevention measures can be taken by having an intervention program that can reduce the risk of this disease. In line with the goals of the New Malaysian government, this study can therefore benefit both universities and employees while also fostering a new generation of workers who are healthier and more productive. It is also possible to implement measurement of non-communicable disease risk factors as part of an ongoing academic program. Additionally, the measurement of non-communicable disease risk factors can also be made into an ongoing program at the university level.

The risk factors of non-communicable disease

The non-communicable disease risk factors are categorized into non-modifiable and modifiable risks. The non-modifiable risk factors include demographic profile, socioeconomic profile, and clinical information. Meanwhile, there are two categories of modifiable risk factors: clinical risk factors (obesity, elevated blood pressure (hypertension), elevated glucose levels (diabetes), and lipids/cholesterol) and behavioral risk factors (smoking, drinking, physical activity, and eating habits). Cardiovascular disease risk scores are evaluated based on the available information such as age, diabetes status, smoking, body mass index, blood pressure, and lipid levels to predict the 10-year risk of cardiovascular disease (Mahmooda et al. 2014).

The non-modifiable risk factors (demographic and socioeconomic profiles, clinical information) and the non-communicable diseases

A study among employees in Iran showed an association between increasing age and men with cardiovascular disease, hypertension, and diabetes (Nahid et al. 2017). These findings are in line with findings on employees in the Ethiopian Urban area (Gebremariam et al. 2018). In Malaysia, old age, men, and Indians are more likely to get diabetes and cardiovascular diseases (Nahid et al. 2017). Those who are married and divorced are said to have more than three times the risk compared to single individuals (Alzeidan et al. 2016). Individuals with low

level of education have a higher risk of getting non-communicable diseases (Tawa 2009). Inequalities in employment and income also affect the prevalence of non-communicable diseases among European employees (Mcnamara et al. 2018). Individuals with comorbidities such as tuberculosis (Peltzer & Africa 2018), diabetes (Bertoluci & Rocha 2017) and hypertension (Leeson 2018) also have a substantial risk of getting non-communicable diseases, especially cardiovascular diseases. A family history of heart disease, diabetes, and hypertension also increases the risk of getting non-communicable diseases, especially if there is a first-class family relationship (father, mother, and siblings) (Alabdouli et al. 2014).

The modifiable risk factors: lifestyle/behavioral factors and the non-communicable diseases

The risk of developing non-communicable diseases can also be affected by lifestyle choices or employee behavior. Several cohort studies have demonstrated evidence of a high-risk relationship between non-communicable diseases and factors like smoking, alcohol consumption, and lack of physical activity (Enkh-oyun & Davaalkham 2016; Khan et al. 2013; Sathish et al. 2017; Yang et al. 2017). In addition, unhealthy eating habits increase the risk of developing non-communicable diseases (Na et al. 2015; Nishida & James 2004). Such circumstances also happened among university employees (Ige et al. 2013, Isfeedvajani et al. 2016). This increase in unhealthy lifestyles is due to stress at work (Manjrekar et al. 2014), workload (Purohit & Varma 2016), and lack of awareness of non-communicable disease risk factors (Manjrekar et al. 2014).

The modifiable risk factors: clinical factors and the non-communicable diseases

Studies at King Faisal University, Saudi Arabia show clinical factors that have a high prevalence among their employees include obesity, increased blood pressure (hypertension), increased glucose levels (diabetes), and increased blood lipids (hyperlipidemia). This study also found that more than 50% of the employees have more than three types of risk factors (Amin et al. 2014). This association is in line with the findings of a study among university staff in Nigeria with 51.8%, 48.5%, 26.7%, 8.0% having hyperlipidemia, hypertension, obesity, and diabetes, respectively (Agaba et al. 2017). A study at Universiti Putra Malaysia also found that the employees have a high prevalence of obesity (76.3%), hypertension (64.0%), and hyperlipidemia (65.5%) (Hayati Kadir Shahar 2015).

The risk factors for cardiovascular diseases

In Malaysia, cardiovascular disease has recorded the highest rate of death with increasing cardiovascular cases each year (Abdullah et al. 2017). A study among employees in Kuala Lumpur showed that 21.8% and 38.9% of them had a high and moderate risk of getting cardiovascular disease (Su et al. 2015). Similar findings were found among patients in Kelantan with 36.2% of them at a high risk for cardiovascular disease (Norhayati et al. 2013). Meanwhile, the Malaysian cohort study showed a 12.7 to 13.2% risk of getting cardiovascular

disease for the next 10 years (Borhanuddin et al. 2018). These scenarios clearly show the increased risk of cardiovascular disease in Malaysia. This is due to the increase of modifiable and non-modifiable risk factors that contribute to the increased moderate and high risk of cardiovascular disease.

Intervention studies and the risk of cardiovascular diseases

Healthy lifestyle interventions are the main key to reducing the risk factors of non-communicable diseases (Bazyar et al. 2017). Previous studies have proven the effectiveness of intervention programs to reduce the risk factors of non-communicable diseases (Arena et al. 2018; Dyson et al. 2015; Krishnan et al. 2018). A health education intervention program using a website was conducted among employees in the Netherlands showed that the intervention program was effective in reducing cardiovascular risk among high-risk workers (baseline: 28.4 ± 7.63; post-intervention: 23.7 ± 10.6, $p<0.05$) (Colkesen et al. 2011). Similarly, a health education intervention program in Japan also showed a decrease in the risk of cardiovascular disease (Zhu et al. 2013). An intervention program conducted on university employees in Atlanta also showed a 12% decrease in cardiovascular risk scores after going through several health education counseling sessions (Al Mheid et al. 2016). The evidence from these studies clearly shows that intervention programs have a positive impact on those at high risk of cardiovascular diseases. Therefore, it would indirectly reduce the burden of treating cardiovascular disease by the government.

Problem statement

The National Health Morbidity Survey report from 2006 to 2015 shows an increased risk of non-communicable diseases such as diabetes, hypertension, hypercholesterolemia, obesity, lack of physical activity, and smoking among Malaysians (Institute for Public Health 2008; Institute for Public Health 2015). This increasing trend is consistent with the high death rate worldwide (63%) caused by non-communicable diseases, particularly diabetes, chronic respiratory illnesses, and heart disease. Approximately 80% of these deaths occur in low- and medium-income countries (WHO 2008). Malaysia is also having an increased number of deaths (73%) due to non-communicable diseases with 68% of them being premature deaths (Yusoff et al. 2013). Therefore, the Ministry of Health Malaysia has prioritized the issue of prevention of non-communicable diseases including their risks in the National Strategic Plan (2016-2025) (Ministry of Health Malaysia 2016). The working population needs to have a low risk of non-communicable diseases to maintain good work productivity. However, previous studies show that working adults are more likely to acquire these risk factors (Gebremariam et al. 2018; Mcnamara et al. 2018). Lack of awareness (Manjrekar et al. 2014), socioeconomic status (Allen et al. 2017), and workload (Purohit & Varma 2016) are among the few factors that have been associated with an increase in non-communicable disease risk factors among working adults. Likewise, university staff are also not an exception as evidenced by previous study findings (Agaba et al. 2017; Alzeidan et al. 2016; Amin et al. 2014). Therefore, it is

crucial for an organization to understand the profile and risk status of non-communicable diseases of the employees so that effective prevention and intervention programs can be implemented in the workplace. Additionally. it can also help to reduce the cost of medical care borne by the government.

Research questions

1. What is the non-communicable disease risk factor profile (demographic, socioeconomic, lifestyle, clinical information, body mass index, blood pressure, blood test, cardiovascular disease risk score) of Universiti Kebangsaan Malaysia (Bangi) staff?
2. How is the non-communicable disease risk factor profile (demographics, socioeconomics, lifestyle, clinical information, body mass index measurements, blood pressure, blood tests, cardiovascular disease risk scores) of Universiti Kebangsaan Malaysia (Bangi) staff between academic and non-academic staff and compared with the general population?
3. What is the relationship between sociodemographic factors, work pressure, work environment, and work performance on the quality of life of Universiti Kebangsaan Malaysia (Bangi) staff?
4. Did the MyMind and SLIM SHAPE 2.0 Intervention Program succeed in lowering cardiovascular disease risk scores, maintaining ideal body weight, and increasing work productivity and quality of life among staff at Universiti Kebangsaan Malaysia (Bangi)?

Research objectives

1. To learn about the profile of non-communicable disease risk factors (demographics, socioeconomic, lifestyle, clinical information, body mass index measurements, blood pressure, blood tests, cardiovascular disease risk scores) of Universiti Kebangsaan Malaysia (Bangi) staff.
2. To compare non-communicable disease risk factor profiles (demographics, socioeconomics, lifestyle, clinical information, body mass index measurements, blood pressure, blood tests, cardiovascular disease risk scores) between academic and non-academic staff of Universiti Kebangsaan Malaysia (Bangi) and with the general population.
3. To analyze the relationship between sociodemographic factors, work pressure, work environment, and work performance on the quality of life of Universiti Kebangsaan Malaysia (Bangi) staff.
4. To determine the effectiveness of the MyMind and SLIM SHAPE 2.0 Intervention Program in lowering cardiovascular disease risk scores, maintaining ideal body weight, and increasing work productivity and quality of life among staff at Universiti Kebangsaan Malaysia (Bangi).

Research hypothesis

1. There are differences in the measurement of non-communicable disease risk factors (demographics, socioeconomics, lifestyle, clinical information, body mass index measurements, blood pressure, blood tests, cardiovascular disease risk scores) between academic and non-academic staff at Universiti Kebangsaan Malaysia (Bangi).
2. There is a relationship between sociodemographic factors, work pressure, work environment, and work performance with the quality of life of Universiti Kebangsaan Malaysia (Bangi) staff.
3. There is a significant decrease in cardiovascular disease scores among the staff of Universiti Kebangsaan Malaysia (Bangi) who participated in the MyMind and SLIM SHAPE 2.0 Intervention Program compared to staff in the control group.
4. There is an increase in work productivity and quality of life among the staff of Universiti Kebangsaan Malaysia (Bangi) who participated in the MyMind and SLIM SHAPE 2.0 Intervention Program compared to staff in the control group.

Research Methodology

This study will be conducted in three main phases; Phase 1 is the baseline measurement, Phase 2 is the intervention phase and Phase 3 is the post-intervention evaluation.

Phase 1 - Baseline

The questionnaire will be developed to cover three projects (First, Second, and Third Projects) where the First Project questionnaire covers non-communicable disease risk factors including demographic profile, socioeconomics, lifestyle (smoking status, alcohol consumption, body mass index, body composition, physical activity, dietary practices) and clinical information (comorbidities, family history of non-communicable diseases). A cross-sectional study will be conducted to determine the non-communicable disease risk factor profile of Universiti Kebangsaan Malaysia (Bangi) staff selected through stratified random sampling. A questionnaire prepared along with anthropometric measurements and blood tests will be disseminated. The results of this risk factor profile will provide a cardiovascular disease risk score value which will serve as the baseline data of the study before the intervention program is conducted. Staff with a Body Mass Index (BMI) of 25 and above will be recruited as participants in one of the MyMind Intervention Program groups, SLIMSHAPE 2.0, or the control group (who will receive a follow-up visit at the UKM Health Centre). The sample size for this cross-sectional study was calculated based on the results of a national survey (National Health Morbidity Survey 2015) with the highest percentage being from lipid levels (47.7%). Using the Kish (1965) formula, the required sample size is 1280 with an expected dropout rate of 40%.

Phase 2 - Intervention

The intervention will be conducted based on Phase 1 findings. The intervention will be implemented together with the Second Research Project (MyMind Intervention Program) and

the Third (SLIM SHAPE 2.0 Intervention Program). The MyMind Intervention Program will introduce the practice of mindfulness, flow at work, and spirituality. Participants will be taught face-to-face in four series of workshops to introduce concepts, activities, exercises, and the use of the MyMind application. An annual calendar will also be provided to help with activities for the 12 weeks. Meanwhile, participants who receive the SLIMSHAPE 2.0 Intervention Program will undergo a weight loss program through seminars, interactive activities, and exercises using the SLIM SHAPE 2.0 module for 12 weeks. Participants in the control group will receive counseling on weight management in terms of nutrition and exercise by the Medical Officer at the UKM Health Centre, Bangi through meeting sessions at the clinic and the provision of printed materials within 12 weeks.

The sample size needed for the intervention program was calculated using Daniel's formula (1999). Based on the intervention study conducted by Ersen et al. (2011), the required sample size is 414 (138 staff for each group) with an expected dropout rate of 30%.

Phase 3 - Post-intervention

Phase 3 is to evaluate the effectiveness of the intervention on the three groups, namely the MyMind Intervention Program, the SLIMSHAPE 2.0 Intervention Program, and the control group. The effectiveness of the intervention will be evaluated based on the reduction of the cardiovascular disease risk score, maintenance of ideal body weight, increase in work productivity, and quality of life between before and after the intervention.

Inclusion criteria:

1. Permanent staff of Universiti Kebangsaan Malaysia (Bangi)
2. Age between 35-60 years old

Exclusion criteria:

1. Temporary staff of Universiti Kebangsaan Malaysia (Bangi)
2. Staff who are on maternity leave
3. Staff who are on sabbatical leave
4. Pregnant staff

Research instrument

The research instrument that will be used in this intervention study is a standardized instrument that has been validated and has good and satisfactory reliability.

1. Questionnaires

The set of questionnaires is divided into seven parts. Part A is the participant's personal and socio-economic information. Part B is the health information of the participant and his/her family. Part C includes information on medicinal consumption, herbs, and alternative treatments. Part D is about the frequency of eating out. Part E includes information on the

consumption of tobacco and related products. Part F is about alcohol consumption information. Part G is about stress. Part H is a proactive personality questionnaire and the last Part I is about the participant's quality of life.

Physical activity will be assessed using the Global Physical Activity Questionnaire (GPAQ-M) Malay version which has been validated and has satisfactory reliability (Soo et al. 2015). Information on dietary patterns will be taken through the Malaysian version of the Structured Dietary History Questionnaire (DHQ) which has also been validated (Suzana et al. 2000).

2. Anthropometry measurement and body composition

Participants' height will be measured without shoes to the nearest 0.1 m using a SECA Bodymeter (Germany) and body weight will be measured to the nearest 0.1 kg using a digital lithium compound scale (Tanita 318, Japan). BMI will be calculated as body weight in kilograms per square of height in meters (kg/m^2). Waist circumference will be measured using a flexible measuring tape to the nearest 0.1 cm. All anthropometric measurements will be measured twice to reduce measurement error using standard methods (ISAK 2006). Measurement of systolic and diastolic blood pressure will be done using a digital measuring device (OMRON brand). Two blood pressure readings will be taken, and the average reading will be calculated. Measurements will be taken by UKM Health Centre staff, Bangi.

3. Blood test

10 ml of blood will be taken by the laboratory staff of Chancellor Tuanku Muhriz Hospital and analyzed at the HCTM laboratory. Blood taken will be used to measure glucose (Fasting Blood Sugar), lipid (Fasting Serum Lipid), kidney function test (Renal Profile), liver function test (Liver Function Test) and haemoglobin A1c.

4. Cardiovascular diseases risk

Cardiovascular disease risk will be assessed using the Framingham coronary disease prediction score. The calculation of this score involves factors of age, total cholesterol level, high-density lipoprotein (HDL) level, blood pressure, diabetes, and smoking factors. This calculation will produce a cardiovascular disease score with more than 20% being high risk and 10-20% being moderate risk and less than 10% being low risk (D'Agostino et al. 2008).

Data analyses

All data will be analyzed using SPSS software version 24. Descriptive data will be presented as frequency (percentage) and mean (standard deviation). Relationships between variables will be analyzed using chi-square tests, t-tests, and correlations. The effectiveness of the intervention program will be analyzed through the MANCOVA test.

Research flow chart

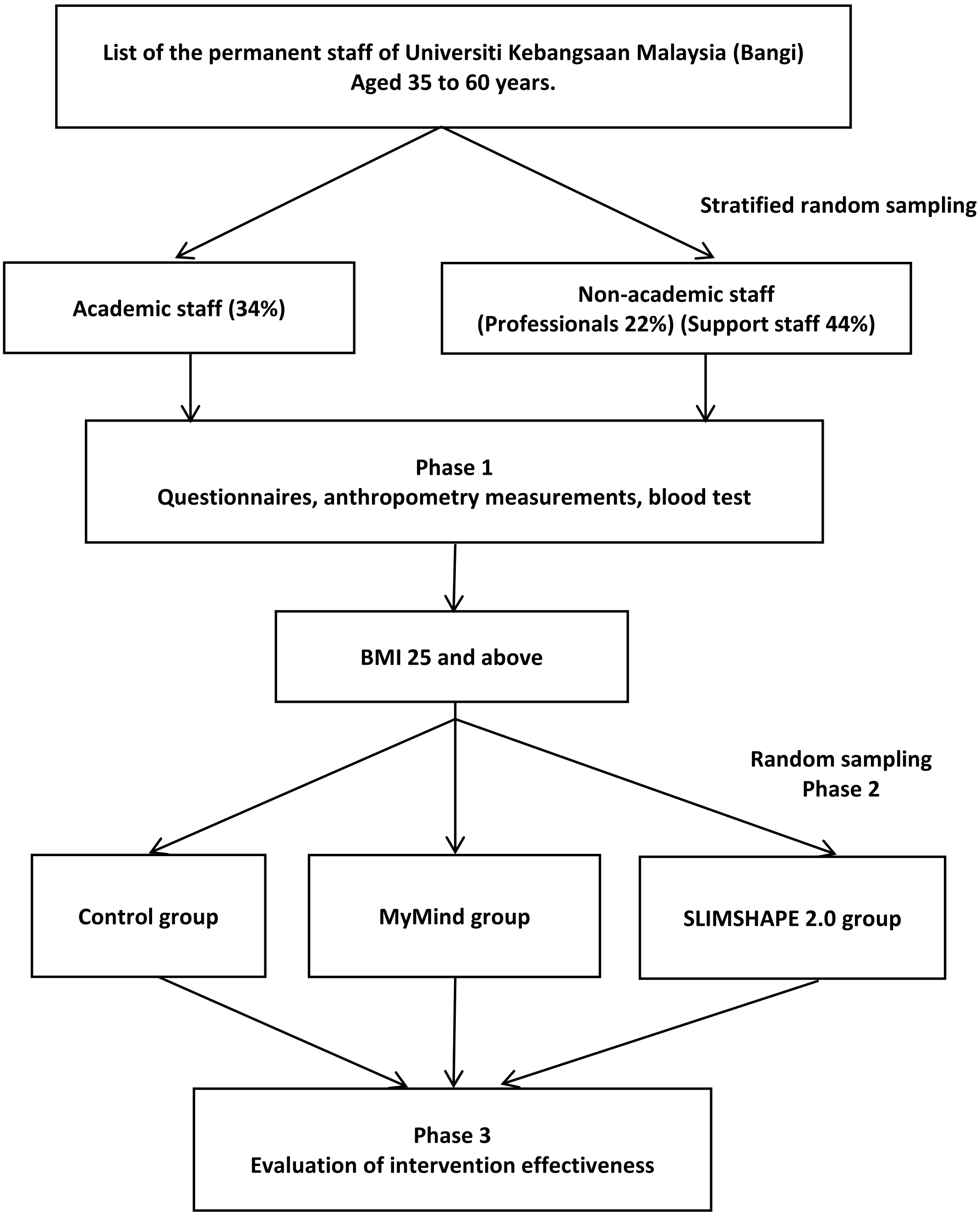

Control group

Participants will receive information about diet, physical activity, and behavior modification for weight loss from Medical Officers at the Universiti Kebangsaan Malaysia Health Centre. Participants will also be given pamphlets on proper diet, exercise, and maintaining a healthy lifestyle.

MyMind intervention group

Participants in the MyMind Intervention Program will be introduced to the practice of mindfulness, flow at work, and spirituality. They will be taught face-to-face in four series of workshops to introduce concepts, activities, exercises, and the use of the MyMind application. An annual calendar will also be provided to assist them with activities for the 12 weeks. Each suggested activity has a duration of five to 30 minutes. Researchers will meet with participants at week 12 and at month 9 to obtain data and participants' views on the MyMind Intervention Program.

List of MyMind intervention activities:

Week	Aktiviti
Week 0	Baseline measurements.<br>Explanation of the baseline findings to the participants
Week 1	Mindfulness workshop (By: Nor Ba'yah and Suzana):<br>✓ Pre-test dan EEG (30 subsample)<br>✓ Mindfulness talk<br>✓ Activity<br>✓ Exercise/Training<br>✓ MyMind Apps<br>✓ Table Calendar
Week 2	Flow workshop (By: Azza and Rusyda):<br>✓ Flow talk<br>✓ Activity<br>✓ Exercise/training
Week 3	Spirituality workshop (By: Arena and Jamiaah):<br>✓ Ceramah Kerohanian<br>✓ Activity<br>✓ Exercise/training
Week 4	Emotional and stress management workshop (By: Nor Ba'yah and Azza):<br>✓ Emotional and stress management talk<br>✓ Activity<br>✓ Exercise/training
Week 5	✓ MyMind Apps (Nor Ba'yah)<br>✓ Experience Method Sampling (notifications)
Week 6	✓ MyMind Apps (Nurul-Azza)<br>✓ Experience Method Sampling
Week 7	✓ MyMind Apps (Rusyda)<br>✓ Experience Method Sampling

Week 8	✓ MyMind Apps (Arena)<br>✓ Experience Method Sampling
Week 9	✓ MyMind Apps (Suzana)<br>✓ Experience Method Sampling
Week 10	✓ MyMind Apps (Jamiaah)<br>✓ Experience Method Sampling
Week 11	✓ MyMind Apps (Rusyda)<br>✓ Experience Method Sampling
Week 12	✓ Post-test<br>✓ Feedback on workshops and Apps

SLIMSHAPE 2.0 intervention group

The group will receive the SLIMSHAPE 2.0 intervention module. The SLIMSHAPE 2.0 module is a SLIMSHAPE module (Rusali et. al 2016) that has been improved with the addition of aspects of mindfulness, flow, and spirituality. The SLIMSHAPE 2.0 Intervention Program will be conducted weekly in a 3-hour session for 12 weeks. The intervention includes discussions, demonstrations, interactive activities, hands-on activities, and training sessions delivered over three months. Lectures related to dietary management and four interactive and hands-on activities related to healthy eating such as calorie counting for food and drinks, healthy cooking demonstrations, a visit to the supermarket, and understanding food labels will all be delivered by a dietitian. Aspects related to physical activity will be delivered through lectures, demonstrations, and group exercise training. These activities will be carried out by sports physiologists and physiotherapists. These sessions aim to empower participants in improving their knowledge, attitudes, and practices towards physical activity and exercise to reduce body weight. In addition to these, participants will also receive mindfulness, flow, and spiritual interventions through Apps called MyMind throughout the intervention program. This application will be developed by members of the research group from the second project (Mindfulness, flow, and spirituality).

List of SLIMSHAPE 2.0 activities:

Week	Aktiviti
Week 0	Parameter measurement at baseline.<br>Explanation of the baseline findings to the participants.
Week 1	Seminar:<br>● Healthy eating and strategy for weight loss by dietitian<br>● Complications of Obesity by Medical Doctor<br><br>Group counseling on diet and nutrition<br>Weight loss target<br>Group exercise
Week 2	Interactive activity:<br>- Calculation of individual calorie needs.

	Group exercise<br>.
Week 3	Seminar:<br>- Mindfulness, flow, and spirituality<br>- Get to know the MyMind Apps<br>Group exercise
Week 4	Interactive activity<br>- Estimating sugar and fat contents in foods and drinks<br>Group exercise
Week 5	Seminar:<br>- Healthy meal preparation<br>Interactive activity:<br>- Healthy cooking demonstration<br>Group exercise
Week 6	Body weight, body composition, and dietary intake measurement (mid-intervention evaluation)<br>Seminar:<br>- Behavioral therapy by Behavioural health expert<br>- Weight loss success story: Follow His Example<br>Group exercise
Week 7	Interactive activity:<br>- Healthy Office Meal pack competition<br>- Understand the Healthy Plate model.<br>Group exercise
Week 8	Interactive activity:<br>- Supermarket tour for education on food labeling and healthy food choices
Week 9	Seminar:<br>- Strategies to overcome weight yo-yo.<br>- Group counseling<br>Group exercise
Week 10	Interactive activity:<br>- Urban farming trip & cooking demonstration<br>Group exercise
Week 11	Seminar:<br>- Review on weight loss target<br>Group exercise
Week 12	Post-intervention assessment

Gantt chart and milestones

Jan 2019 - June 2019: Literature and questionnaire preparation, pilot study and data analysis, updating the intervention module, strengthening good relations with faculty executives at UKM.

April 2019 - June 2019: Phase 1: Baseline measurement and data analysis

July 2019 - June 2020: Phase 2: Intervention (4 months); Phase 3: post-intervention assessment, evaluation of intervention effectiveness, data analysis, and interpretation.

July 2020 - Dis 2020: Final report write-up, manuscript writing for publication, sharing of study findings, and building networks with related agencies.

	2019												2020											
	**Jan**	**Feb**	**Mac**	**Apr**	**May**	**Jun**	**Jul**	**Aug**	**Sep**	**Oct**	**Nov**	**Dec**	**Jan**	**Feb**	**Mac**	**Apr**	**May**	**Jun**	**Jul**	**Aug**	**Sep**	**Oct**	**Nov**	**Dec**
**Preparation of research proposal**																								
**Preparation of questionnaires**																								
**Phase 1 baseline**																								
**Phase 2 intervention**																								
**Phase 3 post-intervention**																								
**Data analysis**																								
**Article write-up**																								
**Final report write-up**																								
**Milestone:**																								
**Ethical approval**	**1/5/2019**																							
**Research activities**	**1/6/2019**																							
**End of data collection**	**1/5/2020**																							
**Final report**	**1/12/2020**																							

References

Abdullah, W. M. S. W., Yusoff, Y. S., Basir, N. & Yusuf, M. M. 2017. Mortality Rates Due to Coronary Heart Disease by Specific Sex and Age Groups among Malaysians. Proceedings of the World Congress on Engineering and Computer Science 2: 1–6.

Agaba, E. I., Akanbi, M. O., Agaba, P. A., Ocheke, A. N., Gimba, Z. M., Daniyam, S. & Okeke, E. N. 2017. A survey of non-communicable diseases and their risk factors among university employees: a single institutional study 28(6): 377–384. doi:10.5830/CVJA-2017-021

Al Mheid, I., Kelli, H. M., Ko, Y. A., Hammadah, M., Ahmed, H., Hayek, S., Vaccarino, V., et al. 2016. Effects of a health-partner intervention on cardiovascular risk. Journal of the American Heart Association 5(10): 1–14. doi:10.1161/JAHA.116.004217

Alabdouli, K., Munir, M. & Soror, M. 2014. Family History of Non-communicable Disease and its Relationship in Acute Coronary Syndrome with or without Diabetes Mellitus. International Journal of Sciences: Basic and Applied Research 16(2): 321–331.

Allen, L. 2017. Are we facing a noncommunicable disease pandemic? Journal of Epidemiology and Global Health 7(1): 5–9. doi: 10.1016/j.jegh.2016.11.001

Allen, L., Williams, J., Townsend, N., Mikkelsen, B., Roberts, N., Foster, C. & Wickramasinghe, K. 2017. Socioeconomic status and non-communicable disease behavioural risk factors in low-income and lower-middle-income countries: a systematic review. The Lancet Global Health 5(3): e277–e289. doi:10.1016/S2214-109X (17)30058-X

Alzeidan, R., Rabiee, F., Mandil, A., Hersi, A. & Fayed, A. 2016. Non-Communicable Disease Risk Factors among Employees and Their Families of a Saudi University: An Epidemiological Study. PLOS ONE 11(11): 1–13. doi: 10.1371/journal.pone.0165036

Amin, T. T., Ibrahim, A., Sultan, A., Mostafa, O. A., Darwish, A. & Al-naboli, M. R. 2014. Profile of Non-Communicable Disease Risk Factors Among Employees at a Saudi University 15: 7897–7907.

Arena, R., Group, A. H. A. W., Guazzi, M., Eacpr, E. S. C., Group, W., Lianov, L., Group, W., et al. 2018. Healthy lifestyle interventions to combat noncommunicable disease — a novel nonhierarchical connectivity model for key stakeholders: a policy statement from the American Heart Association, European Society of Cardiology, European Association for Cardio. European Heart Journal 36: 2097–2109. doi:10.1093/eurheartj/ehv207

Banjare, J. B. & Bhalerao, S. 2016. Obesity associated noncommunicable disease burden. Int J Health Allied Sci 5(81–87). doi:10.4103/2278-344X.180429

Bazyar, F., Gholami-fesharaki, M., Rowzati, M., Steel, M. & Emsc, C. 2017. The relationship between shift work and Framingham risk score: A five-year prospective cohort study Abstract Original Article Shift work (SW) is an unusual working pattern in comparison to the workday. This work pattern is an integral part of the prov. ARYA Atheroscler 13(6): 288–294.

Bertoluci, M. C. & Rocha, V. Z. 2017. Cardiovascular risk assessment in patients with diabetes. Diabetology & Metabolic Syndrome 9(25): 1–13. doi:10.1186/s13098-017-0225-1

Borhanuddin, B., Nawi, A. M., Shah, S. A., Abdullah, N., Zulkifli, S., Zakaria, S., Kamaruddin, M. A., et al. 2018. 10-Year Cardiovascular Disease Risk Estimation Based on Lipid Profile-Based and BMI-Based Framingham Risk Scores across Multiple Sociodemographic Characteristics: The Malaysian Cohort Project 2018.

Chakma, J. K. & Gupta, S. 2017. Lifestyle practice and associated risk factors of noncommunicable diseases among the students of Delhi University. International Journal of Health Allied Sciences 6: 20–25. doi: 10.4103/ijhas.IJHAS

Chung, V. C. H., Lau, C. H., Yeoh, E. K. & Griffiths, S. M. 2009. Age, chronic non-communicable disease and choice of traditional Chinese and Western medicine outpatient services in a Chinese population. BMC Health Services Research 9(207): 1–8. doi:10.1186/1472-6963-9-207

Colkesen, Ferket, Tijssen, Kraaijenhagen & Peters. 2011. Effects on cardiovascular disease risk of a web-based health risk assessment with tailored health advice: a follow-up study. Vascular Health and Risk Management 7: 67. doi:10.2147/VHRM.S16340

D'Agostino, R. B., Vasan, R. S., Pencina, M. J., Wolf, P. A., Cobain, M., Massaro, J. M. & Kannel, W. B. 2008. General cardiovascular risk profile for use in primary care: The Framingham heart study. Circulation 117(6): 743–753. doi:10.1161/CIRCULATIONAHA.107.699579

Dyson, P. A., Anthony, D., Fenton, B., Stevens, D. E., Champagne, B., Li, L., Lv, J., et al. 2015. Successful Up-Scaled Population Interventions to Reduce Risk Factors for Non-Communicable Disease in Adults: Results from the International Community Interventions for Health (CIH) Project in. PLoS ONE 10(4): 1–13. doi: 10.1371/journal.pone.0120941

Enkh-oyun, T. & Davaalkham, D. 2016. A cohort study of chronic diseases for Mongolian people: Outline with baseline data of the Moncohort study. Journal of Epidemiology and Global Health 6(3): 187–196. doi: 10.1016/j.jegh.2015.12.001

Foundation PharmAccess. 2009. Global trends in risk factors for non-communicable diseases.

Gebremariam, L. W., Chi, C., Yatsuya, H. & Haregot, E. 2018. Non-communicable disease risk factor profile among public employees in a regional city in northern Ethiopia (June): 1–11. doi:10.1038/s41598-018-27519-6

Gupta, R. & Xavier, D. 2018. Hypertension: The most important non-communicable disease risk factor in India. Indian Heart Journal 4–11. doi: 10.1016/j.ihj.2018.02.003

Hayati Kadir Shahar. 2015. Prevalence of type 2 diabetes mellitus and its associated factors among a public university staff in Selangor. International Journal of Public Health and Clinical Sciences 1(1): 118–130.
Ige, O., Owoaje, E. & Adebiyi, O. 2013. Non-communicable disease and risky behaviour in an urban university community Nigeria. African Health Sciences 13(1): 4–9.
Institute for Public Health. 2015. National Health and Morbidity Survey 2015 (NHMS 2015). Vol. II: Non-Communicable Diseases, Risk Factors & Other Health Problems. Ministry of Health, hlm. Vol. II. doi:10.1017/CBO9781107415324.004
Institute for Public Health (IPH). 2008. The Third National Health and Morbidity Survey 2006 (NHMS III): Executive Summary.
Isfeedvajani, M. S., Zarchi, A. A. K. & Tavana, A. M. 2016. Diet behavior of Employees at a Medical Sciences University in Tehran, Iran: Iran Health Day 2015. Int J Travel Med Glob Health 4(3): 92–95. doi:10.20286/ijtmgh-040305
Khan, F. S., Lotia-farrukh, I., Khan, A. J., Siddiqui, S. T., Sajun, S. Z., Malik, A. A., Burfat, A., et al. 2013. The Burden of Non-Communicable Disease in Transition Communities in an Asian Megacity: Baseline Findings from a Cohort Study in Karachi, Pakistan. PLOS One 8(2): 1–9. doi: 10.1371/journal.pone.0056008
Khazanah Research Institute. 2017. Death in Malaysia –What are the statistics telling us?
Klemenc-ketis, Z. & Peterlin, B. 2013. Family History as a Predictor for Disease Risk in Healthy Individuals: A Cross-Sectional Study in Slovenia. PLOS One 8(11): 1–5. doi: 10.1371/journal.pone.0080333
Krishnan, A., Ekowati, R. & Baridalyne, N. 2018. Evaluation of community-based interventions for non-communicable diseases: experiences from India and Indonesia. Health Promotion International 26(3): 276–289. doi:10.1093/heapro/daq067
Leeson, P. 2018. Hypertension and cardiovascular risk in young adult life: insights from CAVI. European Heart Journal 19(August): 24–29. doi:10.1093/eurheartj/suw061
Liu, Y., Liu, G., Wu, H., Jian, W. & Wild, S. H. 2017. Sex differences in non-communicable disease prevalence in China: a cross-sectional analysis of the China Health and Retirement Longitudinal Study in 2011. BMJ Open 17(7): 1–7. doi:10.1136/bmjopen-2017-017450
Mahmooda, S. S., Levyb, D., Vasan, R. S. & Wang, T. J. 2014. The Framingham Heart Study and the Epidemiology of Cardiovascular Diseases: A Historical Perspective Syed. Lancet 383(9921): 1933–1945. doi:10.1016/S0140-6736(13)61752-3.
Manjrekar, S. S., Sherkhane, M. S. & Chowti, J. V. 2014. Behavioral risk factors for noncommunicable diseases in working and nonworking women of urban slums. J Midlife Health 5(3): 143–149. doi:10.4103/0976-7800.141220
Mcnamara, C. L., Toch-marquardt, M., Balaj, M., Reibling, N., Eikemo, T. A. & Bambra, C. 2018. Occupational inequalities in self-rated health and non-communicable diseases in different regions of Europe: findings from the European Social Survey (2014) special module on the social determinants of health. European Journal of Public Health 27: 27–33. doi:10.1093/eurpub/ckw223
Ministry of Health Malaysia. 2016. National Strategic Plan for Non-Communicable Disease.
Murphy, S. L., Xu, J., Kochanek, K. D. & Curtin, S. C. 2017. National Vital Statistics Reports Deaths: Final Data for 2015, hlm. Vol. 66.
Na, L., Wu, X., Feng, R., Li, J., Han, T., Lin, L. & Lan, L. 2015. The Harbin Cohort Study on Diet, Nutrition and Chronic Non-Communicable Diseases: Study Design and Baseline Characteristics. PLOS One 10(4): 1–12. doi: 10.1371/journal.pone.0122598
Nahid, K., Babanejad, M., Asadmobini, A. & Karim, H. 2017. The Association of Age and Gender with Risk Factors of Noncommunicable Diseases among Employees in West of Iran. Int J Prev Med 8(9): 1–18.
Nishida, C. & James, W. P. T. 2004. A life course approach to diet, nutrition, and the prevention of chronic diseases. Public Health Nutrition 7(1A): 101–121. doi:10.1079/PHN2003584
Norhayati, M., Sharifah Amirah, S., Lili Husniati, Y. & Mohd Noor, N. 2013. Cardiovascular risk: associated factors, assessment and agreement between WHO/ISH risk prediction chart and Framingham Scoring System among Primary Care Patients in Kelantan, Malaysia. International Journal of Collaborative Research on Internal Medicine & Public Health 5(12): 652–663. Retrieved from http://www.iomcworld.com/ijcrimph/files/v05-n12-02.pdf.

Peltzer, K. & Africa, S. 2018. Tuberculosis non-communicable disease comorbidity and multimorbidity in public primary care patients in South Africa. African Journal of Primary Health Care & Family Medicine 10(1): 1–6.

Perrone-filardi, P., Paolillo, S., Costanzo, P., Savarese, G., Trimarco, B. & Bonow, R. O. 2015. The role of metabolic syndrome in heart failure. European Heart Journal 36: 2630–2634. doi:10.1093/eurheartj/ehv350

Purohit, M. & Varma, K. 2016. Case Report PREVALENCE OF NON-COMMUNICABLE DISEASES IN DOCTORS. International Journal of Innovative Research and Review 4(2): 54–61.

Rusali, R., Wen, L.X., Shahar, S. & Manaf ZA. 2016. Effectiveness of a structured weight management programme at workplace among employees of a petroleum industry in Malaysia. Jurnal Sains Kesihatan Malaysia (Malaysian Journal of Health Sciences). 14 (2): 49-56.

Sardarinia, M., Akbarpour, S., Lotfaliany, M., Bagherzadeh-, F., Bozorgmanesh, M., Sheikholeslami, F., Azizi, F., et al. 2016. Risk Factors for Incidence of Cardiovascular Diseases and All-Cause Mortality in a Middle Eastern Population over a Decade Follow-up: Tehran Lipid and Glucose Study. PLOS One 11(12): 1–16. doi: 10.1371/journal.pone.0167623

Sathish, T., Kannan, S., Sarma, S. P., Razum, O., Sauzet, O. & Thankappan, K. R. 2017. Seven-year longitudinal change in risk factors for non-communicable diseases in rural Kerala, India: The WHO STEPS approach. PLOS One 12(6): 1–9.

Su, T. T., Amiri, M., Hairi, F. M., Thangiah, N., Bulgiba, A. & Majid, H. A. 2015. Prediction of Cardiovascular Disease Risk among Low-Income Urban Dwellers in Metropolitan Kuala Lumpur, Malaysia. BioMed Research International 1–8. doi:10.1155/2015/516984

Suzana S., Earland J. & Suriah A.R. 2000. Validation of a Dietary History Questionnaire against a 7-D Weighted Record for Estimating nutrient intake among rural elderly Malays. *Mal J Nutr* 6: 33 – 44.

Tawa, N. 2009. The prevalence of risk factors for non-communicable diseases among people living in Mombasa, Kenya.

WHO. 2008. Chapter 1 Burden: mortality, morbidity and risk factors.

Yang, F., Qian, D. & Hu, D. 2017. Life-course and Cohort Trajectories of Chronic Non-communicable Diseases Risk Factors in China. Iran J Public Health 46(5): 591–601.

Yusoff, U. N., Mahat, D., Omar, A., Huey, T. C. & Yoep, N. 2013. Burden of Premature Mortality in Malaysia 3(1).

Zhu, B., Haruyama, Y., Muto, T., Yamasaki, A., Bing, Z., Haruyama, Y., Muto, T., et al. 2013. Evaluation of a community intervention program in Japan using Framingham risk score and estimated 10-year coronary heart disease risk as outcome variables: a non-randomized controlled trial. BMC public health 13: 219. doi:10.1186/1471-2458-13-219
