# Supplementary material for: Effect of implementing of the IDEAL discharge model on satisfaction of patient referred to trauma emergency department
Source: PLoS One. 2024 Jun 13;19(6):e0304969. doi: 10.1371/journal.pone.0304969 (PMC11175502; doi:10.1371/journal.pone.0304969)
Supplement: S3 File — (DOC) [file pone.0304969.s005.doc]

	Count	Table N %	Mean	Standard Deviation	
Gender	male	52	60.5%			
	female	34	39.5%			
age			39.14	10.89	
Groupe	intervention	43	50.0%			
	control	43	50.0%			
job1	Employee	16	18.6%			
	Merchant job	37	43.0%			
	housekeeper	24	27.9%			
	Retired	9	10.5%			
marrital	single	24	27.9%			
	married	56	65.1%			
	divorced	2	2.3%			
	Widow	4	4.7%			
Education1	Elementry	7	8.1%			
	High school	11	12.8%			
	Associate Degree	15	17.4%			
	Bachelor's degree	45	52.3%			
	Graduate	8	9.3%			
Economical	Good	14	16.3%			
	Moderate	54	62.8%			
	Low income	18	20.9%			
Teriage_level	3.00	46	53.5%			
	4.00	40	46.5%			
Hospitalization_time			9.10	6.14	
Before 					
Was the department explained to you upon arrival?			1.98	1.14	
Were you delayed when you arrived for a visit?			2.9	4.8	
Was the purpose of the examination explained to you upon arrival?			1.69	.82	
Was the purpose of care explained to you upon arrival?			1.78	.99	
Were people and departments introduced to you upon arrival?			2.0	1.3	
Were the examination results explained to you during hospitalization?			2.0	1.1	
Before doing any procedure, was it explained to you regarding care and procedures during hospitalization?			2.0	1.1	
Did you have unanswered questions during hospitalization?			1.62	1.03	
Was it explained to you about self-care at home?			2.0	1.2	
Was the time of the next visit to the doctor explained to you after discharge?			1.8	1.0	
Were you told how to follow up the laboratory results and...?			2.15	1.09	
Did you have unanswered questions at the time of discharge?			1.70	.88	
Did the nurse have the necessary ability to provide care?			2.35	1.22	
Was the nurse's behavior respectful?			1.83	1.12	
Has the nurse given the necessary information about the continuation of the treatment?			2.17	1.26	
Did the nurse pay the necessary attention to the patients?			1.98	1.14	
Did the doctor give the necessary information about the treatment?			2.09	1.29	
Was the doctor's behavior respectful?			1.70	1.11	
Was the doctor's explanation about the continuation of the treatment complete?			2.21	1.12	
Did the doctor give you the necessary time?			1.79	1.13	
I recommend this treatment center to others.			1.94	1.10	
I am satisfied with the services provided in the emergency department.			2.00	1.11	
After					
Was the department explained to you upon arrival?			3.40	1.17	
Were you delayed when you arrived for a visit?			3.35	1.15	
Was the purpose of the examination explained to you upon arrival?			3.5	1.1	
Was the purpose of care explained to you upon arrival?			3.19	1.19	
Were people and departments introduced to you upon arrival?			2.94	1.26	
Were the examination results explained to you during hospitalization?			3.20	1.29	
Before doing any procedure, was it explained to you regarding care and procedures during hospitalization?			2.51	1.22	
Did you have unanswered questions during hospitalization?			2.67	1.29	
Was it explained to you about self-care at home?			2.74	1.40	
Was the time of the next visit to the doctor explained to you after discharge?			2.73	1.30	
Were you told how to follow up the laboratory results and...?			2.76	1.47	
Did you have unanswered questions at the time of discharge?			2.95	1.43	
Did the nurse have the necessary ability to provide care?			3.1	1.3	
Was the nurse's behavior respectful?			2.27	1.28	
Has the nurse given the necessary information about the continuation of the treatment?			2.78	1.38	
Did the nurse pay the necessary attention to the patients?			2.88	1.29	
Did the doctor give the necessary information about the treatment?			2.76	1.25	
Was the doctor's behavior respectful?			2.57	1.51	
Was the doctor's explanation about the continuation of the treatment complete?			2.52	1.47	
Did the doctor give you the necessary time?			2.85	1.20	
I recommend this treatment center to others.			2.84	1.53	
I am satisfied with the services provided in the emergency department.			3.01	1.36	
On arrival at the emergency department			10.37	5.54	
During hospitalization in the emergency department			5.58	2.41	
Discharge time from the emergency department			7.64	2.76	
Overall satisfaction with nursing care in the emergency department			8.33	3.14	
Overall satisfaction with the doctor in the emergency department			7.79	3.48	
Overall patient satisfaction from the emergency department			3.94	1.72	
On arrival at the emergency department			16.34	3.86	
During hospitalization in the emergency department			8.38	2.43	
Discharge time from the emergency department			11.27	4.12	
Overall satisfaction with nursing care in the emergency department			11.02	3.53	
Overall satisfaction with the doctor in the emergency department			10.70	3.65	
Overall patient satisfaction from the emergency department			5.85	2.25	
total satisfaction first			43.65	12.66	
total satisfaction second			63.56	16.21	
Mean_difference			19.91	20.37	

	Mean	Standard Deviation	
Dimenssion1_After	16.34	3.86	
Dimenssion2_After	8.38	2.43	
Dimenssion3_After	11.27	4.12	
Dimenssion4_After	11.02	3.53	
Dimenssion5_After	10.70	3.65	
Dimenssion6_After	5.85	2.25	


	Mean	Standard Deviation	Minimum	Maximum	
Total_After	63.56	16.21	26.00	90.00	


	�Ñæå	
	ÂÒãæä	
	Mean	Standard Deviation	Minimum	Maximum	
Total_After	77.37 	7.95	58.00	90.00	

	�Ñæå
˜äÊÑá	
	Mean	Standard Deviation	Minimum	Maximum	
Total_After	49.74	8.84	26.00	72.00	
