# Supplementary material for: Effect of implementing of the IDEAL discharge model on satisfaction of patient referred to trauma emergency department
Source: PLoS One. 2024 Jun 13;19(6):e0304969. doi: 10.1371/journal.pone.0304969 (PMC11175502; doi:10.1371/journal.pone.0304969)
Supplement: Attachment — Submitted filename: point to point revised.docx [file pone.0304969.s006.docx]

| **Comments** | **Revised** | **Page** |
| --- | --- | --- |
| 1. Please ensure that your manuscript meets PLOS ONE's style requirements, including those for file naming. The PLOS ONE style templates can be found at |  | - |
| 2. Thank you for stating the following financial disclosure: "No"  Please clarify the sources of funding (financial or material support) for your study.  d) If you did not receive any funding for this study, please state: “The authors received no specific funding for this work.”  Please include your amended statements within your cover letter; we will change the online submission form on your behalf |  | 18 |
| 3. Thank you for stating the following in your Competing Interests section:  "NO authors have competing interests". | Thanks a lot | 19 |
| 4. We note that your Data Availability Statement is currently as follows: "All relevant data are within the manuscript and its Supporting Information files." | We provided the data in word and SPSS files, also provided the questionnaire and content validity result of it. | - |
| 5. We are unable to open your Supporting Information file "Data.sav". Please kindly revise as necessary and re-upload. | We revised it | - |
|  |  |  |
| **Reviewer #1** |  |  |
| 1- Please compare your survey to similar surveys in the literature. | Thanks a lot, We tried to improve it in discussion | 15-17 |
| 2- Please mention the weak and strong points of your study | Thanks a lot, We added it. | 17& 18 |
| 3- I recommend the authors discuss following article in the discussion: I- Sarbazi E, Sadeghi-Bazargani H, Farahbakhsh M, Ala A, Soleimanpour H. Psychometric properties of trust in trauma care in an emergency department tool. Eur J Trauma Emerg Surg. 2023 Aug 21. doi: 10.1007/s00068-023-02348-z. II- Rahmani F, Rezazadeh F, Ala A, Soleimanpour M, Mehdizadeh R, Soleimanpour H. Evaluation of Overcrowding of Emergency Department in Imam Reza Hospital in 2015 by Implementing 2 Scales: NEDOCS and EDWIN. Iran Red Crescent Med J. 2017 June; 19(6):e15609. III- Soleimanpour H, Gholipouri C, Salarilak S, Raoufi P, Vahidi RG, Rouhi AJ, Ghafouri RR, Soleimanpour M. Emergency department patient satisfaction survey in Imam Reza Hospital, Tabriz, Iran. Int J Emerg Med. 2011 Jan 27;4:2. doi: 10.1186/1865-1380-1-2. | Thanks a lot, We use those in references | 20  (ref: 2,3 10) |
| Reviewer 2 |  |  |
| I reviewed the article entitled “Effect of Implementing of the Integrate Discharge Model on Satisfaction of Patient Referred to Trauma Emergency Department” by Kalaiee et al. submitted to PLOS ONE (Manuscript Number: PONE-D-23-29600). In this observational study involving 86 injured patients, the authors mainly investigated that the effect of implementing of the integrate discharge model on patients' satisfaction. They found that implementing of the integrate discharge model was associated with the increased patients' satisfaction, measured by self-reported questionnaire. From these observations, they claimed the usefulness of integrated discharge model in patients with trauma. | Thanks a lot for your mention. |  |
| 1 Much of the abstract is currently spent on background and methods. Please provide in the abstract an informative and balanced summary of what was done and what was found. At the current form, description regarding results is poor, and intervention used in this study is not clear enough. | Thanks a lot, We tried to improve it | 2 |
| 2 This observational study does not follow STROBE Statement Guidelines (<https://www.equator-network.org/reporting-guidelines/strobe/>). The authors should respect the basic rule of scientific writing | Thanks a lot, but our study was a qusi-experimental | - |
| 3 Please indicate the study’s design with a commonly used term in the title, according to the STROBE check list. The title also should be more specific. One example is therefore “Effect of Implementing of the Integrate Discharge Model on Satisfaction of Patient Referred to Trauma Emergency Department: a retrospective observational study at a single medical institution in Iran" Introduction | Thanks a lot, but our study was a quasi-experimental and prospective study. | - |
| 4 At the introduction section, the authors should clarify how the information collected can be used to solve the current problems for trauma. | Thanks a lot, We added it | 5 |
| 5 At the end of the introduction section, please state the any prespecified hypotheses according to the STROBE check list. | Thanks a lot, but our study was a quasi-experimental and use the CONSORT | - |
| Methods 6 Please describe settings and locations more in details (e.g. a tertiary hospital, academic hospital, referral trauma center, number of hospital and ICU beds, and number of annual trauma admission, etc.) where the data were collected. This information should help readers to depict the context of this study more accurately. This reviewer thinks annual trauma volume is especially important because it can greatly affect the quality of trauma care [1, 2], and may be confounding factors in the analyses. | Thanks a lot, We added it | 6 |
| 7 The Ethical approval section should include the relevant date of the approval. | Thanks a lot, We tried to improve it | 18 |
| 8 The authors should describe the intervention used in this study greater in details. As the current form, it is difficult to replicate your study even by other skilled researchers. | Thanks a lot, We tried to improve it | 9-10-11 |
| 9 Please give the characteristic of the data source. How did you assure the quality of data? Since this is an observational study, quality assurance is of vitally important. | Thanks a lot, We tried to improve it | 9-10-11 |
| 10 Study period was just half ot the year (January to July, 2023. Why did you employ such short duration? | Yes, we clarified it and demonstrated it in a figure 1. | 6 |
| 11 Clearly define all exposures, predictors, potential confounders, and effect modifiers. | Thanks a lot, We tried to clarified it in inclusion criteria and homogeneity of groups | 6-8 |
| 12 Describe all statistical methods, including those used to control for confounding. | Thanks a lot, We tried to improve it | 6-8 |
| 13 Describe any efforts to address potential sources of bias, according to the STROBE checklist. For example, blinding is one of the attractive methods to reduce above mentioned biased assessment. If done, please provide who was blinded and how. | Thanks a lot, We tried to clarified it  The researcher who collected data (completed the questionnaire) and the researcher who analyzed the data were blinded | 10-19 |
| 14 Who planned this study, who collected data, and who conducted the statistical analysis? I think if the same researchers are involved in study planning, data collecting, outcome measurement, and statistical analysis, there is a theoretical risk of biased assessment. | Thanks a lot, We tried to clarified it | 10-19 |
| 15 The authors did not provide the information how the questionnaire used here was developed. How did you assure the scientific validity of this survey? As is the current form, the reviewer and readers of this journal cannot judge whether the questionnaire used here is validated or not. This reviewer thinks planning phase is the most important process of the survey that requires detailed description. How did you select the items of your questionnaires? Did you referred to relevant studies conducted in other countries or other setting when developing the questionnaire? In addition, the authors should provide the rationale of outcome measurements. | Thanks a lot, We tried to clarified it | 9 |
| 16 Please provide the rationale of the items included in this survey more in details. | Thanks a lot, We tried to clarified it |  |
| 17 Please provide an English version of the full questionnaires used in this study to facilitate readers' understanding regarding this project. | We provided it as supplementary file |  |
| 18 The authors should describe relevant dates, including periods of questionnaires development, questionnaires distribution, follow up, and collection. | We describe it. | 6 |
| 19 Please explain how missing data were addressed. Did authors use complete dataset? | We describe it. | 6 |
| 20 A flow diagram reporting the numbers of individuals at each stage of study—eg numbers potentially eligible, is missing. | We describe it. | 6 |
|  |  |  |
| 22 Table 1. Baseline characteristics of the participants Many vital information is missing. Give characteristics of study participants such as vital sings including GCS score, blood pressure, and respiratory rate, need of endotracheal intubation and mechanical ventilation, Charlson Comorbidity Index, trauma etiology (penetrating or blunt), Abbreviated Injury Scale of each body parts, TRISS based probability of survival etc. At this current form, many readers including myself find it difficult to image the characteristics of study subjects. In addition, these variables would have confounded the results. There are too many unmeasured confounders. This is the serious flaw of your manuscript. The authors should adjust for such important confounders to provide more reliable data. | We describe it. | 7 |
| 23 Much of the discussion section is simply the list of previous studies, restate or rephrase the results and background that have already described in results and introduction section. Most parts of discussion are too speculative, and not based on the data obtained in this study. This reviewer thinks the discussion is not thought evoking one. In addition, the discussion section should indicate how the findings of this study can be used to solve the current problems. How and for what do we use these results presented here to improve the current trauma care and why? | Thanks a lot, We tried to improve it | 3-4 |
| 24 The limitation section needs substantial revision. Please discuss limitations of the study, taking into account sources of potential bias or imprecision. Consider the important limitations and do not just list them but consider their relevance and how they might bias the results. Discuss both direction and magnitude of any potential bias. | Thanks a lot, We tried to improve it | 17 |
| 25 What is the strength of this observational study? Please indicate after the limitation section. | Thanks a lot, We tried to improve it | 16-17 |
| 26 Please discuss the generalizability (external validity) and potential clinical implications for practice of the study results | Thanks a lot, We tried to improve it | 16-17 |
| 27 Please indicate future research direction more in details, immediately after limitation section. | Thanks a lot, We tried to improve it | 16-17 |
| Conclusion 28 The conclusion section is just rephrased of the observed findings. The study implications are not shown in the conclusion section. How to use this conclusion to improve the clinical outcome of the patients? Please indicate. | Thanks a lot, We tried to improve it | 2-17 |
| Minor points |  |  |
| 29 Keep abbreviations to a minimum. Do not use non-standard abbreviations unless they appear at least three times in the text. | Thanks a lot, We tried to improve it | - |
| 30 The authors should provide the minimal anonymized data set used in this manuscript, according to the journal's policy. | Thanks a lot, We tried to improve it | - |
| **Reviewer #3:** |  |  |
| 1. The main problem of this manuscript is the ambiguity in the sampling method, the method of randomly assignment of samples to control and test groups. | Thanks a lot, We tried to improve it | 5-6 and 10 |
| 2. When and by whom were the samples selected? | Thanks a lot, We tried to clarified it | 10- 19 |
| 3. The formula for calculating the sample size is not clear and numbers have not been placed in it? | Thanks a lot, We tried to clarified it | 7-8 |
| 4. The numerical value of alpha and sigma is not mentioned. Considering the nature of the emergency department and the short stay of patients, has it been possible to train them? | Thanks a lot, We tried to clarified it | 7-8 and 11 |
| 5. What was the role of caregivers in this study and why was it not included in the statistical analysis? | Thanks a lot, We tried to clarified it | 5 |
| 6. Also, this manuscript needs basic revision in terms of written English language. In table number 1, the sum of control and test samples is not correct in some parts. | We tried to improve it | - |
| 7. Finally, many of the references are old, and if possible, newer references should be used instead them. Reference number 16 does not have the year of publication. | Thanks a lot, We tried to improve it  We revised that Reference (number 24) and added its year 2021 | 21-23  22 |
| 8. The reference writing format style needs to be edited. | Thanks a lot, We tried to improve it | 21-23 |
