# Supplementary material for: Effect of implementing of the IDEAL discharge model on satisfaction of patient referred to trauma emergency department
Source: PLoS One. 2024 Jun 13;19(6):e0304969. doi: 10.1371/journal.pone.0304969 (PMC11175502; doi:10.1371/journal.pone.0304969)
Supplement: Attachment — Submitted filename: Point to point.docx [file pone.0304969.s007.docx]

**Point-to-point responses to comments**

Dear Reviewer

Thanks a lot for your mention and valuable comments. We tried to improve our manuscript.

Best regards.

| **Comments** | **Revised** | **Page** |
| --- | --- | --- |
| Reviewer #4: Abstract: Further review by an English native speaker is needed. | Thanks a lot  We tried to improve it. | - |
| e.g. "Eighty-size patients were participated" should be Eighty-six patients participated. and this sentence should mention the patients were recruited between these dates, rather than all patients involved the whole period. | Thanks a lot.  We corrected it | Page 2 |
| Some methods are specified in the results section, include independent t-test in the methods (but shouldn't it be a paired t-test as each patient has a pre and post value? Please specify the statistical methods used and confirm they were appropriate. | Thanks a lot.  We tried explaining it.  We added a pre-test result.  We added a paired T test result. | 13-15  Page 13  Page 15 |
| Methods pg 5: why was it a quasi-experimental interventional study? Isn't it an interventional study? | Thanks a lot.  To prevent the dissemination of information between intervention and control groups, we first collected the control group data and then the intervention group date. | - |
| line 111: 3,0000 is not the standard way a number is presented: do you mean 30,000? | Thanks a lot.    We corrected it | Page 6 |
| Power calculation: what is the difference of 10 between. Clarify if it in the mean overall score between groups at the discharge visit? you state in the text d=10 unites, but then on line 133 it is 1.25. Please clarify. line 135 has too many equals signs, and how does 40/14 = 40? | Thanks a lot.    We corrected it | Page 7  Page 8 |
| line 140: matched: this means different things statistically and in a sampling manner.  How did it work - the matching - was it used to recruit particular participants in the second group? Was it a coincidence that both groups went from 50 to 43? i.e. did recruitment stop when the 43rd patient completed study assessments? | This was really a coincidence. At the time of admission, patients were selected based on the inclusion criteria, and they were trained at the time of discharge. Data was collected before and after education. At first, we collected the control group data, but data collection stopped after 50 people were selected, and 43 people were recruited. Then, for the intervention group, we stopped data collection after 43 people returned. | - |
| pg 11 - the first paragraph seems to be explaining the acronym IDEAL - the EAL is clear, but what about the I and D? | Thanks a lot.    We explained it | Page 11 |
| line 233 and 237 and 241: should this be p>0.05? units for time in emergency room? | Thanks a lot.    We corrected it | Page 12-13 |
| table 3: is satisfaction of the emergency department made up of the sum of the other 6 or 7 elements? This needs to be clearer in the methods section, and state that this is the primary endpoint, secondary tests are done for individual components of the score. I had thought the primary quantity of interest was the satisfaction at discharge time (after the intervention had occurred in the intervention group). Please clarify for all readers. | Thanks a lot.  We tried to correct it.  The first rows was total score, we removed it from table and mention total score in text. | Page 14-15  Table 3 and 4 |
