# Supplementary material for: Effect of implementing of the IDEAL discharge model on satisfaction of patient referred to trauma emergency department
Source: PLoS One. 2024 Jun 13;19(6):e0304969. doi: 10.1371/journal.pone.0304969 (PMC11175502; doi:10.1371/journal.pone.0304969)
Supplement: Attachment — Submitted filename: Point to point second.docx [file pone.0304969.s008.docx]

**Point-to-point responses to comments**

Dear Editor and Reviewer

Thanks a lot for your mention and valuable comments. We tried to improve our manuscript.

Best regards.

| **Comments** | **Revised** | **Page** |
| --- | --- | --- |
| **Editorial comment** | | |
| Please review your reference list | Thanks a lot  We reviewed the reference list, deleted 6 and 12, and revised the others. | 21-24 |
| **Reviewer 4 comment** | | |
| line 241 in track changed version: what is the unit for time in the emergency room | Thanks a lot  We added it (hours). | 12 |
